# I. Retention of Fœtal Skeleton. II. Paint-Poisoning1Read before the Illinois State Veterinary Medical Association, November 16, 1900.

**Published:** 1900-12

**Authors:** W. H. Welch

**Affiliations:** Lexington, Ill.


### REPORTS OF CASES.

### I. RETENTION OF FŒTAL SKELETON. II. PAINT-POISONING.[1]

By W. H. Welch, M.D.C.,
LEXINGTON, ILL.

Case I.—Retention of Fœtal Skeleton. The subject was a registered Scotch collie bitch, four years of age. She was presented for the operation of ovariotomy, with the following history :

Previously, at the ages of one and two years, she had given birth to two healthy litters of puppies. The first litter numbered five, while the last consisted of eight, all strong and vigorous. Nothing unusual at this time was noticed, but before the puppies were weaned she became greatly emaciated, which, to the owner's mind, was readily accounted for by the excessive drain upon her system, as a consequence of nursing her young. This emaciated condition, a rough coat, together with a very capricious appetite and fits of vomiting, were maintained for a period of perhaps a year.

I was at this time consulted in regard to her, and as the owner was the proprietor of quite a large kennel, which subsisted almost entirely on baked oatmeal-mush for a ration, the case was considered one of indigestion. The administration of tonics, together with a complete change of diet, soon resulted in restoring her to perfect health, with the exception that during the two years she did not come in heat.

Shortly after she was disposed of to a farmer and sheep-raiser, who, desiring to be rid of the annoyance of rutting, presented her for operation.

The patient, after a twenty-four hours' fast, is securely muzzled and held by two assistants on a clean oil-cloth. The hair is clipped along the median line, and the field of operation thoroughly washed with carbolized water. An incision is made through skin and linea alba, beginning close to the umbilicus and extending backward an inch in length. Then, puncturing through the peritoneum, search for the Fallopian tubes begins. Astonished at not being able to locate the same, the incision is extended backward until it is dis-

[1] Read before the Illinois State Veterinary Medical Association, November 16, 1900.

covered, and, drawing out the right ovary, it is removed. Now, following back the right cornua, with a view of locating the left, the uterus is reached, when it is noticed that it is much smaller than normal, being indurated and firmly contracted upon some hard substance within its walls. A probe passed through the vagina into the os uteri determines it to be of a calcareous nature. Proceeding up the left cornua, a similar substance is met within an inch or so from the ovary.

Removal of the remaining ovary, together with the entire uterus, was now decided upon, and easily accomplished by means of the écraseur.

Examination showed that within the left Fallopian tube were the cranial bones of a fœtus, while the uterus proper contained the remaining bones of the skeleton, and was so firmly contracted on them as to be diminished to about one-half its natural size.

During the night, in spite of the administration of proper stimulants, the patient succumbed from the effects of the operation. No doubt the extent of the incision through the parietes, which was at least two inches in length, aided such result. Could the existing condition have been known, and the incision have been made at a point posterior, or the operation been performed through the flank, it is barely possible that it might have resulted successfully.

The case is interesting as illustrating tubal pregnancy, with decomposition and disintegration of the softer tissues and retention of the bony skeleton for a period of two years, with the animal apparently enjoying good health.

CASE II.—PAINT-POISONING. On May 18, 1898, I was called to the country to see some milch-cows that were said to be scouring and very sick.

Upon arrival at the farm, I found two of the three cows owned by the farmer scouring very profusely. Both were in a recumbent position, very weak, and when compelled to rise, which they did with great reluctance, staggered and seemed to have but little control of the organs of locomotion. Pulse nearly imperceptible, and greatly increased in frequency. Temperature a trifle subnormal. The mucous membranes of eyes injected and eyes weeping. Exhibited some pain, as evidenced by a grunt accompanying each respiration.

I was informed by the owner that the first symptom of ailment was on the previous evening, when, instead of giving their usual supply of milk, the lacteal secretion was completely suppressed.

In the morning it was seen that they had been scouring profusely through the night.

About a week previous to this they had been changed from one pasture into an adjoining one. A careful survey of both pastures and the roadside, to which they had access upon two different occasions, failed to offer a satisfactory theory. The water which they were drinking was from the same tank and well.

A few days previous to the commencement of the trouble one of them was seen to drink from an old gallon paint-keg, which had been thrown on the wood-pile, and into which the water from a recent rain had fallen. This had been at once removed, and nothing more thought of regarding it. Upon examination it was found to contain about an inch of white lead, covered with linseed oil, but which now appeared perfectly dry. Upon being questioned as to this being a probable cause, the opinion was ventured that it was hardly probable, as the symptoms did not appear to bear out that theory.

They were placed on stimulants combined with opiates, but upon the following morning, as the diarrhœa still continued and they appeared no better, consultation was asked for, and Dr. Alverson was summoned.

After receiving a history of the cases and making a careful examination of the patients, he concurred in the opinion that it was due to the ingestion of some substance the nature of which we were unable to decide.

The following night one of the two died. A post-mortem held the next morning revealed extensive inflammation of all four divisions of the stomach, together with slight enteritis. Nothing, however, was disclosed as to the etiology.

On May 21st, however, imagine the owner's surprise when on going to the stable he found both his remaining cows lying dead, one of which had given no notice of illness.

Dr. Alverson was again summoned, and together a post-mortem was held on each of the two cows. Each showed about the same general inflammation that No. 1 exhibited, but the contents of the stomach of No. 3 were dry and impacted. In each of them among the ingesta were found particles of dried paint consumed in drinking from the keg.

---

The Kansas City Veterinary College opened its winter session on September 14, 1900, with the largest attendance in the history of the college.

